# Non‐Innocent Ligands as Mediators for Visible‐Light‐Initiated Element–Carbon Bond Homolysis in Main Group Chemistry

**DOI:** 10.1002/anie.202507060

**Published:** 2025-07-07

**Authors:** Jonas O. Wenzel, Johannes Werner, Frank Breher

**Affiliations:** ^1^ Institute of Inorganic Chemistry (AOC) Karlsruhe Institute of Technology (KIT) Engesserstraße 15 76131 Karlsruhe Germany

**Keywords:** Main group chemistry, Non‐innocent ligands, Photochemistry, Radical chemistry, Redox active ligands

## Abstract

Photoinitiated homolysis of element–carbon bonds is an important method for the generation of carbon‐centered radicals in catalysis and organometallic or polymer chemistry. In this respect, the use of earth‐abundant main group elements such as aluminum or silicon is attractive. Generally, subvalent species derived from these typically redox‐inactive elements are unstable and within their high‐valent configuration +III (Al) or +IV (Si) comparatively strong E─C bonds are formed. Therefore, E─C homolysis usually requires shortwave UV irradiation, which hampers their use as radical sources. Some reports in the literature show that visible‐light‐induced E─C homolysis is possible when a redox non‐innocent ligand (NIL) is coordinated to the organometallic fragment. In a simplified view, the NILs provide chromophoric moieties, which can absorb energy in form of light and subsequently convert it to break the element–carbon bonds. The resulting main group element radicals are in turn stabilized by delocalization of the unpaired electron, effectively lowering the dissociation energy of the E─C bond. In this article, the effects of NILs as mediators for visible‐light‐induced E─C bond homolysis in main group chemistry are discussed on the basis of selected literature reports, and future opportunities and challenges are highlighted.

## Graphical abstract

This minireview discusses the facilitation of E─C bond homolysis of main group compounds with visible light by the coordination of redox non‐innocent ligands. Important conceptual coherencies, potential applications, and future challenges are highlighted with respect to selected literature reports.

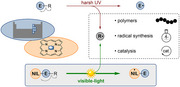

## 1 Introduction and Concept

**Scheme 1:**
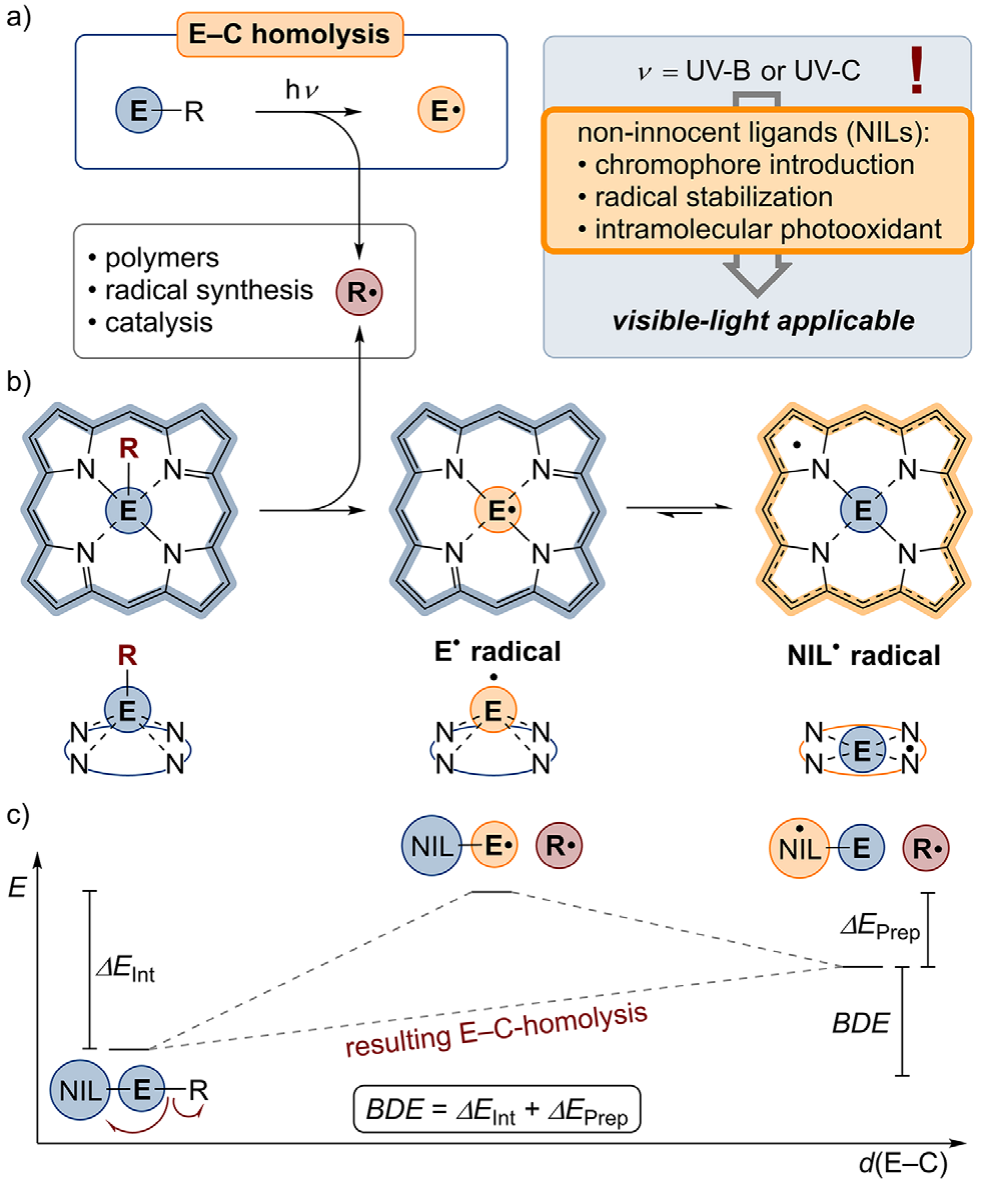
a) Photo‐induced E–C bond homolysis (E = light s‐ or p‐ block element, R = carbon substituent). b) E─C bond homolysis within a porphyrin scaffold as exemplarily non‐innocent ligand (NIL) and schematic depiction of electromeric structures of the radical photolysis product. c) Schematic energy scheme for the homolysis of E─R bonds and influence of E^•^ radical stabilization (BDE < 0, *ΔE*_Int_ < 0, *ΔE*_Prep_ > 0 by definition).

Carbon‐centered radicals are highly reactive transient intermediates^[^ ^1^, ^2^ ^]^ in radical organic synthesis,^[^ ^3^ ^]^ transition metal catalysis,^[^ ^4^, ^5^, ^6^, ^7^, ^8^ ^]^ or radical polymerization.^[^ ^9^, ^10^, ^11^ ^]^ The photoinitiated homolysis of M─C bonds within organometallic compounds is an important mode of carbon radical formation (Scheme 1a).^[^ ^12^ ^]^ Such homolyses are well known as elementary reactions of transition metal catalysis that proceed via radical pathways, such as carbene‐transfer^[^ ^13^ ^]^ or C─C coupling reactions.^[^ ^14^, ^15^ ^]^ Moreover, photoinitiated M─C homolysis enables the utilization of organometallic compounds as Type I photoinitiators in polymer chemistry.^[^ ^16^, ^17^ ^]^ Ideally, M─C photolysis is triggered by visible light (400–780 nm), which enhances the probability of a selective photoreaction and reduces UV‐induced health concerns.^[^ ^14^, ^18^, ^19^, ^20^ ^]^ Moreover, it is desired to use organometallics of cheap and earth‐abundant elements,^[^ ^21^, ^22^ ^]^ which are often less connected to geopolitical imbalances.^[^ ^23^ ^]^ These characteristics are fulfilled by many of the “lighter” main group elements,^[^ ^24^, ^25^ ^]^ as for instance silicon and aluminum are the second and third most abundant elements in the earth crust.^[^ ^26^ ^]^ In general, p block element‐based photoinitiators represent a vivid field of research,^[^ ^17^ ^]^ which is clearly demonstrated by the great interest in acyl germanium^[^ ^27^, ^28^, ^29^ ^]^ or phosphorus compounds^[^ ^30^, ^31^, ^32^, ^33^, ^34^, ^35^, ^36^ ^]^ in radical polymer chemistry.

Organometallics providing a stable homolysis product are prone to carbon radical formation, yielding a subvalent element (E)‐ or metal (M)‐centered radical. Thus, higher stability of element radicals results in lower E─C or M─C bond dissociation energies (BDEs). This is often found for transition metals,^[^ ^15^, ^16^, ^37^, ^38^, ^39^, ^40^, ^41^, ^42^ ^]^ wherefore homolytic reactivity is observed thermally^[^ ^43^ ^]^ or triggered by UV‐A, UV‐B, or visible light,^[^ ^15^, ^16^, ^37^ ^]^ giving polymerization photoinitiators such as the homoleptic neopentyl (Np) compounds Ti(Np)_4_^[^ ^44^ ^]^ and Cr(Np)_3_,^[^ ^45^ ^]^ as well as Cp_2_TiMe_2_,^[^ ^46^ ^]^ or the commercially available Irgacure 784 [Cp_2_TiAr_2_; Ar = 2,6‐difluoro‐3‐(1*H*‐pyrrol‐1‐yl)phenyl].^[^ ^47^ ^]^ Also compounds of “heavy” p block elements with a principle quantum number of *n* > 4 (e.g., In, Tl, Sn, Pb) show smaller BDEs^[^ ^48^ ^]^ and E─C bonds are photolyzed more easily.^[^ ^49^, ^50^, ^51^, ^52^ ^]^ Thus, elements of d and lower p block share higher stability of low oxidation states, but do not benefit from the high abundancy and low costs found for “lighter” main group elements.^[^ ^26^ ^]^ Additionally, organometallic compounds of tin, lead, or thallium are connected to severe toxicity, making their application less attractive.^[^ ^53^, ^54^, ^55^, ^56^ ^]^ Classical organometallic compounds require harsh UV irradiation for photoinduced cleavage of Li─C (e.g., Ph─Li),^[^ ^57^, ^58^, ^59^ ^]^ B─C (e.g., BEt_3_),^[^ ^60^, ^61^, ^62^, ^63^, ^64^, ^65^, ^66^, ^67^ ^]^ Al─C (e.g., AlMe_3_),^[^ ^68^, ^69^, ^70^, ^71^, ^72^, ^73^, ^74^, ^75^, ^76^ ^]^ Ga─C (e.g., GaMe_3_),^[^ ^72^, ^77^, ^78^ ^]^ or Si─C (SiMe_4_)^[^ ^79^ ^]^ bonds (Table 1). The necessity of using UV irradiation for homolytic reactivity originates from both the lack of a chromophore and comparatively strong E─C bonds^[^ ^48^, ^80^, ^81^ ^]^ due to highly energetic subvalent species of elements like Al or Si.^[^ ^82^, ^83^ ^]^ It is noted that pronounced stability of the organic leaving group induces weaker E─C bonds,^[^ ^84^, ^85^, ^86^, ^87^, ^88^ ^]^ but for conventional carbon moieties, the values from Table 1 are suggested as good reference points. The energy required for photochemical bond cleavage reactions is generally high for main group elements of the third or fourth period.^[^ ^52^, ^89^, ^90^, ^91^, ^92^, ^93^, ^94^, ^95^ ^]^

**Table 1:** Experimental bond dissociation energies (BDE(E─C)_exp._, in kJ mol^−1^ or nm) of selected main group compounds; corresponding wavelength (λ in nm) of reported photoinduced E─C bond cleavage reactions.

Compound	BDE(E–C)_exp._		λ
	(kJ mol^−1^)	(nm)	(nm)
BMe_3_	366.1^[^ ^81^ ^]^	326.8	–^a)^
AlMe_3_	263.2^[^ ^81^ ^]^	454.5	190–270^[^ ^71^, ^72^, ^73^ ^]^
GaMe_3_	240.6^[^ ^81^ ^]^	497.2	190–290^[^ ^72^, ^77^ ^]^
InMe_3_	162.8^[^ ^96^ ^]^	743.8	190–310^[^ ^72^ ^]^
TlMe_3_	114.7^[^ ^97^ ^]^	1043.0	–
SiMe_4_	329.7^[^ ^81^ ^]^	362.8	175–193^[^ ^79^ ^]^
GeMe_4_	288.7^[^ ^98^ ^]^	414.4	193^[^ ^99^ ^]^
SnMe_4_	223.0^[^ ^81^ ^]^	536.4	193^[^ ^100^ ^]^
PbMe_4_	150.2^[^ ^81^ ^]^	796.4	255–275^[^ ^49^ ^]^

^a)^ Hg‐sensitized photolysis of BEt_3_ accomplished with 193 nm.^[^ ^61^ ^]^

The generation of carbon‐centered radicals via E─C bond photolysis of abundant p block elements by visible light is a formidable challenge in modern main group chemistry. Despite the fact that “pure” main group organometallics require shortwave UV irradiation, various reports from the last 50 years can be found in which compounds with redox non‐innocent ligands (NILs)^[^ ^101^, ^102^, ^103^, ^104^, ^105^, ^106^ ^]^ undergo E─C photolysis by visible‐light excitation. The coordination of NILs to organometallic entities has two important consequences. On the one hand, the chromophoric systems of NILs are introducing π–π* transitions into organometallic complexes, which followingly absorb light at lower wavenumbers compared to bare organometallics with only σ–σ* transitions.^[^ ^107^ ^]^ As described in following sections, those redshifted π–π* transitions are potentially connected to reactivity of the E─C‐bond. On the other hand, main group radical species are stabilized by electron density delocalization into the ligand scaffold,^[^ ^108^, ^109^, ^110^ ^]^ leading to lower BDEs of the E─C bond. This can be elucidated by a simple thought experiment. If an E─C bond, flanked by a NIL (Scheme 1, NIL–E–R), is critically elongated until radicals form, without structurally and electronically altering the remaining molecule (Scheme 1b,c), the required energy is comparable to the BDE of the “pure” organometallic fragment with only small electron density delocalization into the ligand. Hereby, an element‐centered radical (i.e., [NIL–{E^•^}]) is formed. In the presence of NILs, the existence of another electromer^1^ is reasonable, which is best described as a ligand‐centered radical with high valency at the central atom (i.e., [{NIL^•^}–E]). The equilibrium [NIL–{E^•^}] ⇌ [{NIL^•^}–E] depends on the redox properties of both the central element and the NIL itself. If the electromer [{NIL^•^}–E] is thermodynamically favored, the hypothetical molecule [NIL–{E^•^}] is conceptually relaxing geometrically and electronically into the ground state geometry of [{NIL^•^}–E]. This relaxation, which is coextensive with the storage of the unpaired electron in the NILs’ π system, resembles a photooxidation of the E─C bond by the NIL and a gain in energy compared to a sole homolytic E─C bond cleavage. In such cases, the effective BDE is lowered by the NIL. This can also be expressed within the formalism of the energy decomposition analysis (EDA),^[^ ^111^, ^112^, ^113^ ^]^ in which the BDE is separated into the interaction energy (*ΔE*_Int_) between both fragments [NIL–{E^•^}] and {R^•^} and the preparation energy (*ΔE*_Prep_), which accounts for the electronical and structural reorganization of [NIL–{E^•^}] to [{NIL^•^}–E].

This article highlights the general concept of enabling E─C bond homolysis with visible light by coordinating NILs to a lighter main group element. Although the present concept is, in principle, adaptable to other E─X bonds (e.g., X = halides or chalcogenides),^[^ ^114^, ^115^ ^]^ the title review focuses on the generation of carbon‐centered radicals. The topic is clearly underrepresented in the corresponding literature because overall reports about visible‐light photolysis of E─C bonds are still scarce and often discussed within the scope of highly varying contexts and different fields of chemistry. This interdisciplinary nature is why this article elaborates the main concept by reference to selected literature reports without claiming comprehensiveness.

## 2 Pertinent Examples

### 2.1 Main Group Porphyrins

**Scheme 2:**
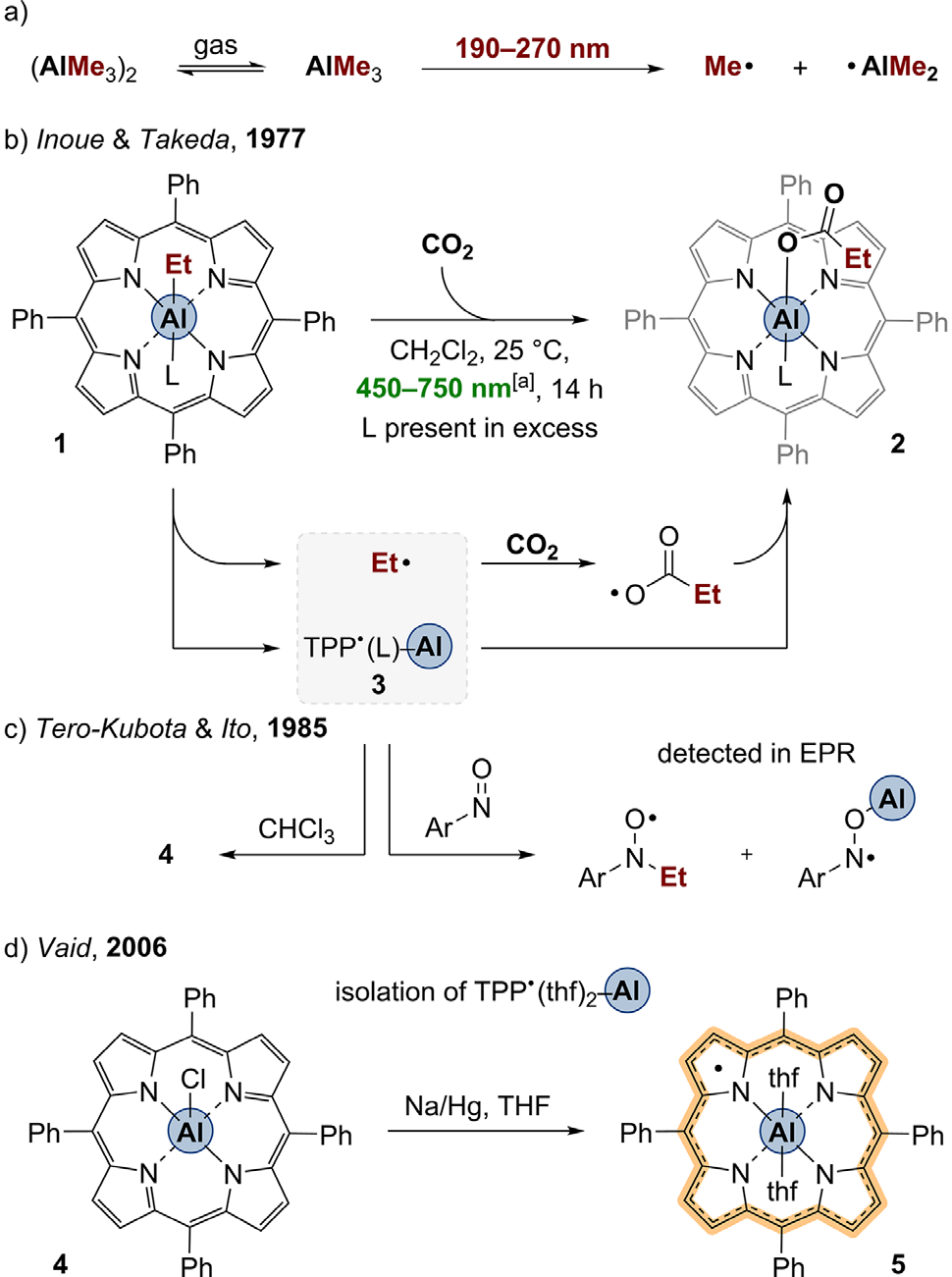
a) UV–photolysis of trimethyl aluminum. b) First observation of the visible‐light homolysis of the Al─C bond in [TPP–Al(L)Et] (**1**) during the CO_2_ activation. c) Trapping experiments of the ethyl radical and homolysis product **3**. d) Synthesis of the [{TPP^•^}–Al(thf)_2_] radical **5** (L, 1‐methylimidazol; ^[a]^irradiation using a Xe‐lamp with cutoff filters below 450 and above 750 nm).

Porphyrinoid ligands are by far the dominating ligand class in mediating visible‐light‐induced homolytic E─C bond cleavage in main group compounds.^[^ ^116^, ^117^, ^118^ ^]^ Very early findings of the present concept were reported by Inoue et al. in the late 1970s as part of their investigation on the influence of varied nitrogen‐containing organic molecules as Lewis bases on the reactivity of organometallics with carbon dioxide. It was reported that the ethyl‐substituted aluminum tetraphenylporphyrin (TPP) complex **1** reacts faster with CO_2_ to the corresponding aluminum propionate **2** when the reaction is conducted under visiblelight (450–750 nm) rather than in the dark (Scheme 2b).^[^ ^119^ ^]^ Furthermore, the presence of 1‐methylimidazole was mandatory for the observed reaction with CO_2_ indicating that the Lewis acid–base adduct represents the reacting species. Later, the same group observed visible‐light accelerated Michael addition of the ethyl moiety of **1** to vinyl ketones.^[^ ^120^ ^]^ In 1985, Tero‐Kubota and Ito proposed that visible light induces the homolytic cleavage of the Al─C bond in aluminum porphyrin complexes based on spin trapping experiments and electron paramagnetic spin resonance (EPR) spectroscopy (Scheme 2c).^[^ ^121^ ^]^ The authors furthermore described reaction quantum yields in the magnitude of 10^−3^ for varied excitation wavelengths in the visible‐light region, but with the highest reaction rates for excitation within the Q bands at 568 and 612 nm. Thus, photolysis of **1** induced by visible‐light irradiation yields the ethyl radical and the radical species [{TPP^•^}–Al(L)] (**3**). Electrophilic substrates such as CO_2_ or electron deficient alkenes (e.g., acrylates) are then inserted into the Al─C bond via a radical reaction mechanism. When **1** is irradiated in CHCl_3_ without any of the above‐mentioned substrates, the compound is converted to [TPP–AlCl] (Scheme 2c, **4**).^[^ ^121^ ^]^ Interestingly, no decomposition nor photoreactivity was observed when the irradiation was conducted in benzene solution. It appears reasonable to assume that the homolysis is still proceeding in aromatic solvents, but that the recombination is faster than the reaction of the organic radical with the solvent (in contrast to the reaction in halogenated solvents). In 2006, Vaid reported on the isolation of [{TPP^•^}–Al(thf)_2_] (**5**) by reduction of [TPP–AlCl] (**4**) with Na/Hg (Scheme 2d).^[^ ^122^ ^]^ Apart from the octahedral coordination environment around Al, cyclic voltammetry and EPR spectroscopic studies revealed that **5** is a ligand‐centered radical without actual subvalent or radical character on the aluminum atom, which is still in the formal oxidation state +III. This preservation of high valency is often found for reduced aluminum species within the framework of redox NILs.^[^ ^123^, ^124^, ^125^ ^]^

Note that the presence of donor molecules (e.g., THF) influences the equilibrium between different electromers promoting high valency.^[^ ^108^, ^126^, ^127^ ^]^ Still, it seems reasonable that **5** is a prototype for and electronically similar to the primary photolysis product **3**. It is assumed that the radical delocalization into the porphyrin scaffold stabilizes the primary homolysis product thermodynamically.^[^ ^123^ ^]^ The effective BDE of the Al─C bond is conclusively suggested to be below ∼260 kJ mol^−1^, i.e., the typical BDE of conventional aluminum organometallics (cf. Table 1). This stabilization facilitates the homolytic Al─C bond cleavage. Together with the introduction of the porphyrin chromophore this leads to photoreactivity of aluminum porphyrin complexes initiated by wavelengths up to 612 nm, which corresponds to an energy input of 195 kJ mol^−1^. For comparison, wavelengths of up to 270 nm (443 kJ mol^−1^) are required for the photolysis of the chromophore‐lacking AlMe_3_ (cf. Scheme 2a).

The reason for Al─C bond weakening upon excitation of **1** on a photophysical level is only little understood. In 1989, Rohmer published ab initio calculations on the double‐configuration self‐consistent field (DC‐SCF) level of theory concerning the photophysical processes happening during Al─C homolysis in [TPP–AlMe] (**6**).^[^ ^128^ ^]^ As expected from traditional organoaluminum chemistry, the Al─C bond is polarized but shows pronounced covalent character. With large Al─C distances of 500 pm, namely dissociation, radical separation into [TPP–{Al^•^}] and {CH_3_^•^} is favored over an ionic dissociation into [TPP–Al]^+^ and [CH_3_]^−^. It is postulated that excitation of **6** with visible light correlates with π–π* transitions of the porphyrin scaffold and that the photoreaction takes place from the first excited singlet state or from an energetically accessible triplet state. Furthermore, conical intersections^[^ ^129^ ^]^ between different singlet as well as between different triplet surfaces were mentioned as very likely. Sophisticated transient spectroscopic measurements or state‐of‐the‐art computational investigations with respect to the multiconfigurational character that would specify those yet diffuse mechanistic postulates have been, to the best of our knowledge, not reported yet. Despite the lack of comprehensive understanding of the underlying processes, applications of the homolytic photochemistry of alkyl‐substituted aluminum porphyrins were developed as for instance in CO_2_‐activating homogeneous catalysis^[^ ^130^ ^]^ and in systems for living polymerizations of acrylates.^[^ ^131^, ^132^, ^133^ ^]^

**Scheme 3:**
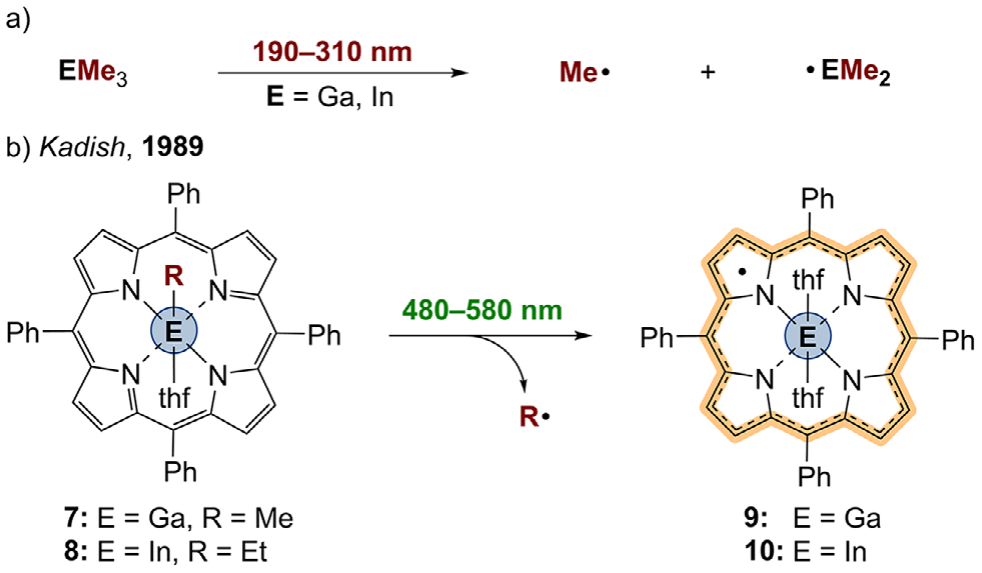
a) UV–photolysis of trimethyl gallium or indium. b) Visible‐light‐photolysis of the E─C bond within gallium and indium porphyrins.

Photoinduced E─C homolysis is likewise occurring in alkyl‐substituted gallium^[^ ^134^, ^135^ ^]^ and indium^[^ ^134^, ^136^, ^137^, ^138^, ^139^ ^]^ porphyrin complexes, despite the photolysis of conventional gallium and indium organometallics requires harsh UV light (Scheme 3a).^[^ ^72^, ^77^ ^]^ During irradiation of compounds such as [TPP–GaMe] (Scheme 3b, **7**) or [TPP–InEt] (**8**), E─C bond homolysis afford the alkyl radicals {Me^•^} or {Et^•^}, respectively. Bonds to vinyl and alkynyl moieties did not undergo any photolysis. According to electrochemical investigations as well as EPR and UV–vis spectroscopic studies, porphyrin radicals **9** and **10** are formed upon irradiation, similar to the previously described findings for **1**.

The remaining question to be asked is how electronic excitation of the π system of the ligand can lead to dissociative modes of the central E─C bond. Based on transient UV–vis spectroscopy and Stern–Volmer triplet quenching experiments with ferrocene or trinitrofluorenone (TNF), Kadish postulated that **7** and **8** are photoreacting from the triplet state.^[^ ^134^ ^]^ But the connection between excited state dynamics and electronic structure still seems obscure for porphyrin complexes of group 13 elements.

**Scheme 4:**
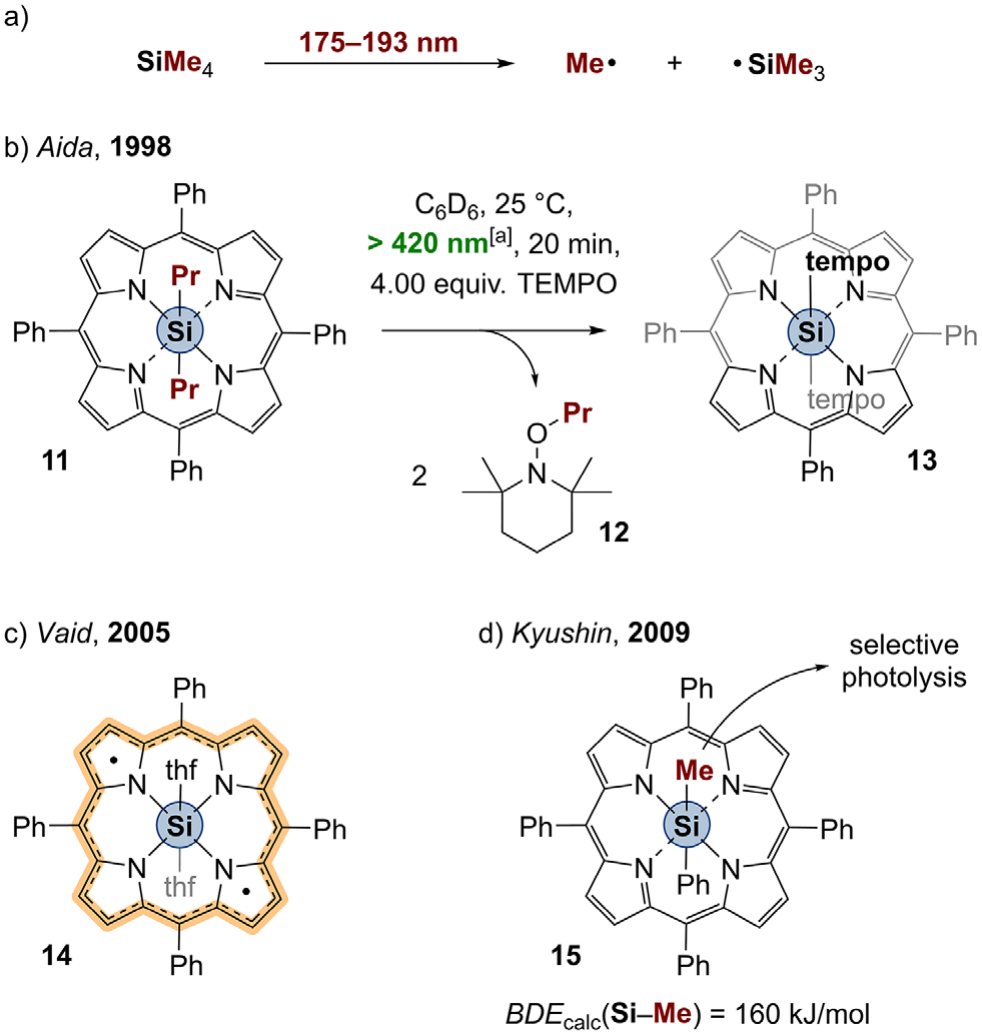
a) UV–photolysis of SiMe_4_. b) Radical trapping experiments during the Si─C homolysis of silicon porphyrins (^[a]^xenon arc lamp used). c) Isolated [{TPP^••^}–Si(thf)_2_] diradical **14**; d) selective Si─Me bond cleavage.

More sophisticated studies were undertaken for porphyrin compounds of group 14. Visible‐light‐induced E─C bond cleavage was reported in the case of E = Si,^[^ ^140^, ^141^, ^142^, ^143^ ^]^ Ge,^[^ ^144^, ^145^, ^146^, ^147^ ^]^ and Sn^[^ ^147^, ^148^, ^149^ ^]^ In 1998, Aida reported on the Si─C homolysis of [TPP–SiPr_2_] (Scheme 4b, **11**).^[^ ^140^ ^]^ In the presence of tetramethylpiperidine‐*N*‐oxide (TEMPO) as a radical trap, the adducts TEMPO–Pr (**12**) and [TPP–Si(tempo)_2_] (**13**) were detected underlining the radical reactivity. If TEMPO was added after irradiation of **11**, no formation of the aforementioned radical adducts were described unless the mixture was irradiated again. The authors explained this by postulating a ligand‐centered diradical [{TPP^••^}–Si] species as homolysis product, which is too inert for thermal reactivity with TEMPO, but regains reactivity upon excitation. Seven years later, Vaid isolated the THF‐adduct [{TPP^••^}–Si(thf)_2_] **14** (Scheme 4c) supporting Aidas postulate of ligand‐centered diradical [{TPP^••^}–Si].^[^ ^150^ ^]^

In silicon as well as in germanium porphyrins, functional groups with sp^2^ or sp‐hybridized carbon atoms showed much lower quantum yields and, thus, slower homolysis.^[^ ^140^, ^144^ ^]^ It was shown that in the heteroleptic complex [TPP–Si(Me)Ph] (**15**), the methyl moiety can be selectively homolyzed leaving Si─Ph bond fully intact (Scheme 4d).^[^ ^141^ ^]^ As part of this selectivity study, frontier Kohn–Sham orbitals were calculated for **15** and significant contribution of the Si─C bonds was described. The excitation was postulated to depopulate the highest occupied molecular orbitals (HOMOs) and decrease electron density within the Si─C bonds. This means that the central Si─C bond is involved in hyperconjugation with the π system of the ligand and actively participating in frontier orbital interactions. This is why the excitation of the π system is inevitably connected to electronic influence and weakening of the central E─C bond. Furthermore, the authors calculated the BDE of the Si─Me bond in **15** to amount to 160 kJ mol^−1^. According to that, the coordinating porphyrin ligand reduces the BDE dramatically compared to BDEs of typical Si─C bonds (330 kJ mol^−1^ in SiMe_4_, cf. Table 1), which are usually photolyzed by harsh UV light (Scheme 4a).^[^ ^79^ ^]^ Compound **15** therefore nicely illustrates the concept of E─C bond weakening by NILs.

The idea of weakening the Si─C bond by removing electron density from the bonds is consistent with the observation of radical formation as a consequence of one‐electron oxidation of E─C bonds,^[^ ^151^ ^]^ which is, for instance, applied with silicates^[^ ^152^, ^153^, ^154^, ^155^ ^]^ or borates^[^ ^156^, ^157^, ^158^, ^159^ ^]^ in photoredox catalysis. In the case of the title compounds, the NILs play the role of an intramolecular photooxidant.

### 2.2 Dipyrromethenes

**Scheme 5:**
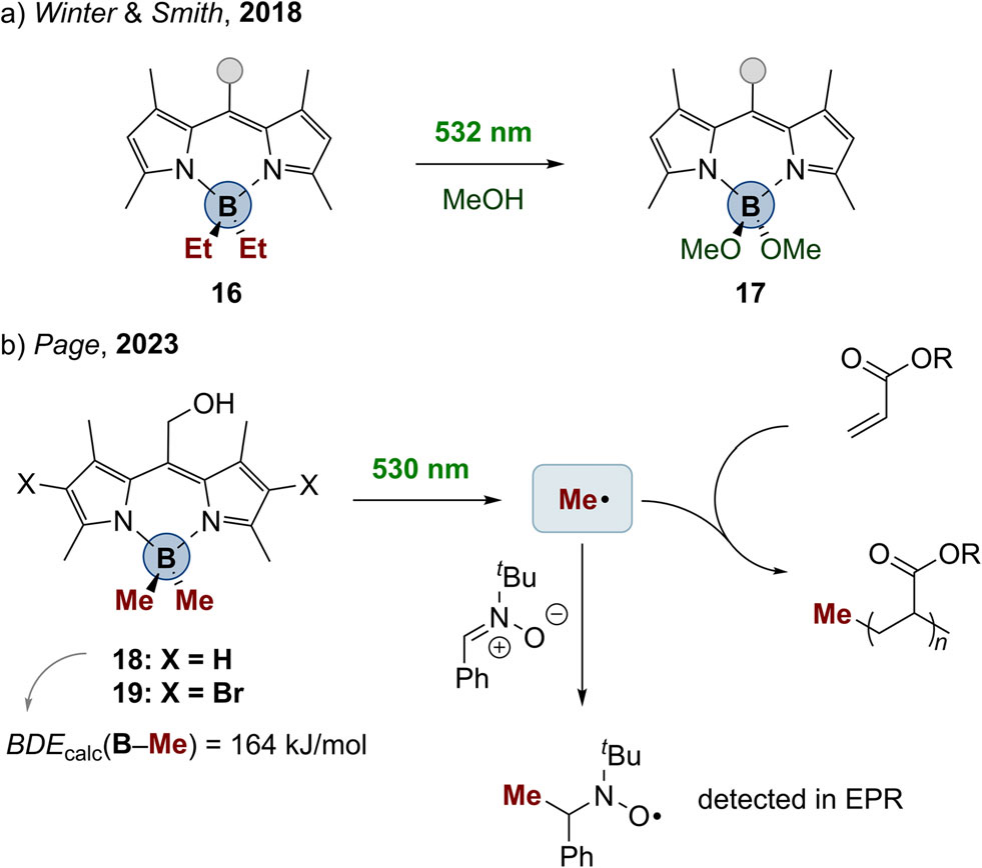
a) Visible‐light‐induced methanolysis of boron dipyrromethene complexes. b) Visible‐light‐initiated homolysis of B─C bond in dipyrromethene complexes and trapping of the methyl radical (R, isobornyl).

Interestingly, also dipyrromethenes, which can be seen as “half a porphyrin”, give rise to compounds with photoreactivity of E─C bonds. In 2018, Winter and Smith reported the methanolysis of **16** to **17** upon irradiation in methanol (Scheme 5a).^[^ ^160^ ^]^ While compound **16** with a B─C bond was only weakly fluorescent but photoreactive, the B─O counterpart **17** was photostable and highly fluorescent. This reaction resembles visible‐light‐enabled alcoholysis of aluminum porphyrins,^[^ ^161^ ^]^ but the authors did not directly describe radical reactivity as it was not the focus of their study. Just recently, Page discussed the radical nature of this kind of photochemistry.^[^ ^162^ ^]^ The authors described very efficient polymerization of acrylates to poly(acrylates) by irradiation with visible light, for example, with 530 nm in the case of compound **18** (Scheme 5b). They supported radical B─C bond cleavage by spin trapping the methyl radical with the aid of *N*‐*tert*‐butyl‐α‐phenylnitrone (PBN) and subsequent characterization of the adduct by EPR spectroscopy. The B─C BDE in compound **18** was calculated to amount to 164 kJ mol^−1^, which is again significantly smaller compared to BMe_3_ (366 kJ mol^−1^, cf. Table 1).

The peripheral bromination of the ligand scaffold provided compound **19** (Scheme 5b). Interestingly, this derivative showed slightly redshifted absorptions and the monomer conversion during photoinitiation was 16 times faster as compared to **18**. Transient UV–vis absorption spectroscopy revealed for **18** and **19** long‐living excited states with lifetimes in the magnitude of 10–30 µs, i.e., typical values for triplet states. The fluorescence quantum yield was 0.39 for **18** and 0.09 for **19**, respectively. This corroborates that the boron dipyrromethene complexes by Page homolyze from the triplet state after excitation. Therefore, this homolysis can be enhanced by shifting the excited state channel branching more toward intersystem‐crossing to the triplet surface by heavy‐atom‐effects.^[^ ^163^ ^]^ The substance class of boron dipyrromethenes is usually known from the highly fluorescent borondifluoride dipyrromethenes (BODIPYs).^[^ ^164^ ^]^ Also, heavier analogs of BODIPYs were reported to show pronounced fluorescence.^[^ ^165^ ^]^ It is noted that the introduction of E─C bonds to any ligand scaffold can change the excited state dynamics completely. In the case of BODIPYs, the exchange of B─F by B─C bonds drastically lowers the fluorescent quantum yields and additionally gives rise to E─C photoreactivity in the excited triplet state.^[^ ^162^ ^]^ The homolytic photoreactivity is likewise diminished by the exchange of the B─C by B─O bonds in **16** (Scheme 5a), but fluorescence is turned on resulting in **17** as strong emitter.^[^ ^160^ ^]^ Therefore, the concept presented in this article is also important to consider when E─C bond‐containing fluorescence emitters are designed. The group of Kretschmer, for instance, reported about highly fluorescent aluminum complexes that are emitting from the first singlet excited state but show dissociative character of the Al─C bonds within the second singlet excited state.^[^ ^166^ ^]^

### 2.3 Phthalocyanines

**Scheme 6:**
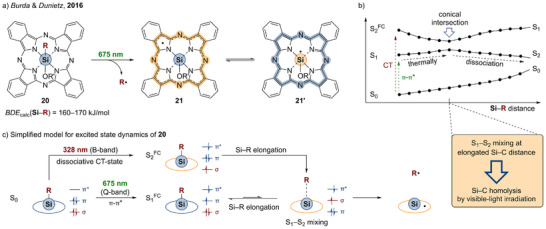
a) Schematic representation of the Si─C bond homolysis of the silicon phthalocyanine **20** (R = (CH_2_)_3_SH, R’ = SiMe_2_{(CH_2_)_3_NMe_2_)}; b) schematic representation of the surface scan of **20** redrawn with permission from Reference 169. Copyright 2016 American Chemical Society; c) simplified schematic model of the excited state dynamics of **20**.

Similar to porphyrins, the visible‐light‐homolysis of Si─C bonds was also reported for compounds with phthalocyanine ligands (Pc).^[^ ^167^, ^168^ ^]^ The silicon phthalocyanine system [Pc−Si(R)OR'] **20** was mechanistically thoroughly investigated by Burda and Dunietz.^[^ ^169^ ^]^ Upon irradiation of **20** with 675 nm, the EPR spectrum indicates the formation of ligand‐centered radicals [{Pc^•^}−Si(OR“)] **21** (Scheme 6a). The BDE of the Si─C bond was calculated to 170 kJ mol^−1^ by using the polarizable continuum model (PCM) solvent correction with MeOH or 160 kJ mol^−1^ in the gas phase. Also, in this instance, the relevant BDE is lowered by radical stabilization through electron density delocalization. The authors computed the spatial distribution of the singly occupied molecular orbital (SOMO) of the radical primary homolysis product **21**. Accordingly, the unpaired electron is indeed delocalized within the NIL π system, but still some spin density remains at the central silicon atom. Thus, both electromers [{Pc^•^}−Si(OR”)] (**21**) and [Pc−{Si^•^(OR')}] (**21′**) shown in Scheme 6a are suggested to be of significance, at least in the absence of donor molecules. By time‐dependent density functional theory (TDDFT) computations, the first singlet excited state S_1_ of **20** (Q‐band) was characterized with exclusive π–π* character. The energy of this state upon Si─C stretch within a relaxed potential energy surface scan slightly increases indicating its nondissociative nature.

In contrast, the second excited singlet state S_2_ (B band) showed charge transfer (CT) character. The energy of this CT state decreases with elongation of the Si─C bond starting from the vertical Franck–Condon geometry (S_2_^FC^) to a molecular structure, for which conical intersection and energetic inversion of the CT and π–π* state is observed (S_1_–S_2_ mixing, Scheme 6b). Within the excited CT state, the polarization of the Si─C bond is inverted compared to the electronic ground state being consistent with its dissociative nature. Thinking in localized orbitals, the described phenomenon can also be expressed simplified as follows (Scheme 6c): Initial excitation of **20** leads to a π–π* transition of the phthalocyanine. Afterwards, an electron transfer from the Si─C bond into the SOMO takes place, which is related to a stretch of the Si─C bond and a small energy barrier of 40 kJ mol^−1^. The one‐electron oxidation of the Si─C bond leads to dissociation, providing carbon‐ and phthalocyanine‐centered radicals, respectively. The authors stated that there were neither relevant nor energetically accessible excited triplet states. Together with the sophisticated studies on the triplet reactivity of **18** and **19**, this highlights the variety of possible photochemical reaction pathways and the complex excited state dynamics responsible for E─C photolysis.

## 3 On the Impact of π Acceptor Properties

**Scheme 7:**
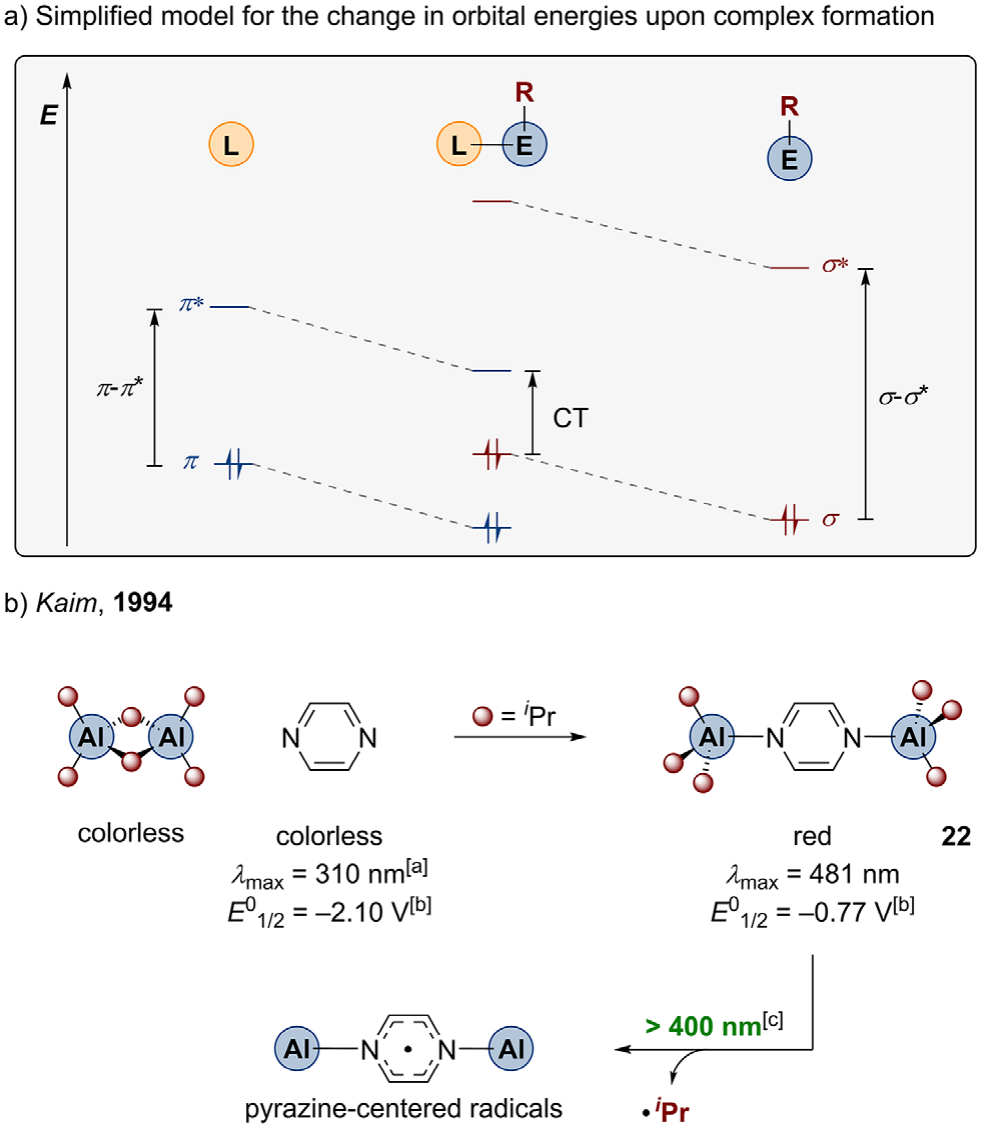
a) Qualitative schematic molecular orbital diagram to illustrate the changes in orbital energies upon coordination of a NIL to an organometallic entity. b) Reaction between (Al*^i^* Pr_3_)_2_ and pyrazine gives the charge‐transfer complex **22**, which undergoes visible‐light homolysis of the Al─C bond (^[a]^taken from the literature^[^ ^179^ ^]^; ^[b]^versus standard calomel electrode (SCE); and ^[c]^Hg‐lamp with cutoff filter^[^ ^171^ ^]^).

The above mentioned computations concerning silicon porphyrins and phthalocyanines imply that the frontier orbitals of photoactive compounds show significant coefficients at the breaking Si─C bond.^[^ ^141^, ^169^ ^]^ This leads to the assumption that E─C bonds with larger contribution to the HOMO provide higher CT character of the first absorptions and more efficient photolytic reactions. This concept can be more clarified on the basis of studies by Kaim et al., who investigated complexes of main group organometallics and six‐membered N‐heterocyclic ligands.^[^ ^170^, ^171^ ^]^ During coordination of any σ‐donating ligand to Lewis acidic and coordinatively unsaturated organometallic main group entities, the orbital energies of both the ligand and the organometallic fragment are mutually influenced. Due to the loss of electron density, the π orbitals of the ligand are energetically stabilized, whereas the increasing electron density around the main group element lifts the E─C σ‐bonding orbitals.^[^ ^172^ ^]^ This very simplified interaction scheme is depicted in Scheme 7a. The HOMO of NIL‐coordinated organometallic fragment can either possess π or σ character depending on the relative energies of the former π and σ levels of the separated entities. But the complex is nevertheless characterized by a smaller HOMO–LUMO gap compared to the separated fragments. In this simplified view, electronic excitation within the first optical absorption of the complex is denoted as CT state, in which electron density is redistributed from the E─C bond forming σ orbitals into π* orbitals of the ligand. The extent of this redistribution, and thus the CT character, depends on the σ character of the HOMO. The wavelength of the first optical transition is obviously dependent on the relative energies of the HOMO and the LUMO of the complex.^[^ ^173^ ^]^ Irradiation within the CT band can trigger the E─C bond homolysis. Also, other absorptions than the HOMO–LUMO transitions can be connected to photoreactivity as lower‐lying molecular orbitals can likewise possess significant coefficients involving the E─C bond.

The discussed correlations can be illustrated using the example of the complexation of Al*^i^* Pr_3_ with pyrazine (Scheme 7b).^[^ ^171^ ^]^ Both precursors are colorless compounds showing no absorptions in the visible‐light range. The complex **22** is, in contrast, a dark red compound showing an absorption maximum at 481 nm. The electronic transition of lowest energy was characterized as CT transition during which molecular orbitals mainly located at the Al─C bonds are depopulated, whereas the electron density in π* orbitals of the pyrazine is increased. Irradiation of **22** with visible light (Hg lamp with cutoff filter > 400 nm) results in the homolytic cleavage of Al–C bonds and the formation of pyrazine‐centered radicals. The energetic lowering of the pyrazine π levels upon coordination is underlined by the change of the reduction potentials of pure pyrazine (*E* ^0^_1/2_ = −2.10 V versus SCE) compared to **22** (*E* ^0^_1/2_ = −0.77 V versus SCE). The extent of changing the orbital energies of the ligand and the organometallic fragments upon complexation depends on different factors such as the electron affinity of the main group atom, the denticity of the ligand, and the orbital energies and symmetries. Some metal fragment/ligand combinations lead to very small HOMO–LUMO gaps, for which single‐electron transfer (SET) can already occur without the need of light. This can be seen as limiting case of the principle shown in Scheme 7a and is observed during many reactions of organometallics with electron‐poor organic molecules.^[^ ^174^, ^175^, ^176^, ^177^, ^178^ ^]^ The opposite limiting case of a poor acceptor ligand is the photoreactivity of NIL‐lacking prototypical organometallics, which need UV light for photolysis, as they possess only σ* orbitals of high energy as acceptor orbitals.

**Scheme 8:**
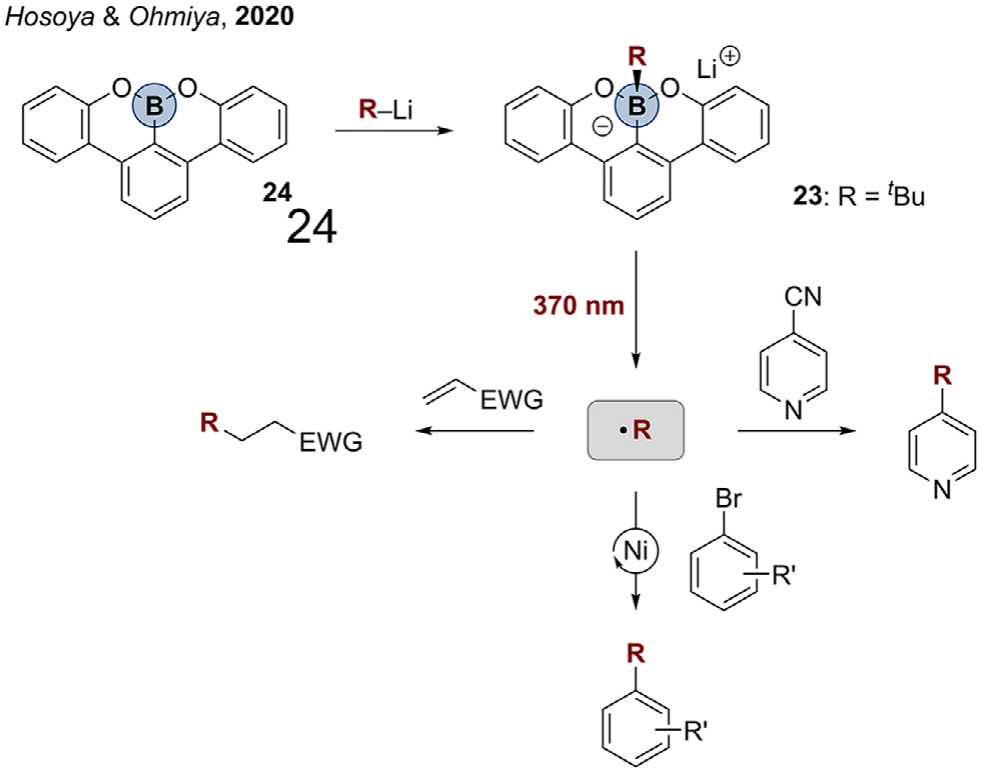
Radical generation from alkylborate **23** and following synthetic utilization of the organic radical.

In between plain organometallics and NIL containing complexes presented in this article are compounds with more electron‐rich ligands, whose non‐innocence is less obvious and whose photoreactivity is triggered by light between shortwave UV and visible light. An interesting example of B─C homolysis with a comparatively electron‐rich ligand was published in 2020 by Hosoya and Ohmiya (Scheme 8).^[^ ^180^ ^]^ The absorption maximum of the boracene alkyl borate **23** was determined to 370 nm, which does not fall in the visible‐light region, but initiated B─C photolysis. The wavelength required for the photoreactivity of **23** compared to the so far presented examples supports the idea of shorter wavelengths necessary in the case of more electron‐rich NILs. The authors showed the potential of **23** in C─C coupling reactions like Giese‐type alkylations or decyanoalkylations of electron deficient arenes. Furthermore, they used **23** as nucleophilic reagent in Nickel‐catalyzed photo‐Suzuki‐type couplings, highlighting the potential of E─C homolytic reactivity in transition metal catalysis.

At the present time, it is uncertain whether CT character of the excited state is a necessary requirement for homolytic E─C bond cleavage. However, it is proposed that compounds that show CT absorptions are prone to photolysis. As discussed already for the phthalocyanine **20**, the dissociative CT does not necessarily correspond with the first excited state accessible by visible light. The correlation between the extent of the CT character and the photolysis quantum yield is still unclear and remains a subject for future studies.

It is concluded that two criteria are important for the photolysis of E─C bonds and, followingly, for the choice of the NIL while designing organometallic complexes that should undergo visible‐light photolysis. On the one hand, frontier orbital coefficients at the E─C bond of interest influence the extent of dissociative character of electronic states. On the other hand, the excitation energy is the energetic gap between the frontier orbitals involved in the electronic transition. Accordingly, NILs with pronounced π acceptor properties and E─C bonds with pronounced donor properties lead to compounds, which undergo photolysis at comparatively long wavelengths. Still, the accepting character of NILs has to be in the correct range to make the CT accessible by visible light rather than UV, but also not too pronounced to induce thermal electron transfer chemistry. Two methods can help to estimate whether a main group complex with a given NIL undergoes visible‐light photolysis: 1) The potential difference (*ΔE* = *E*_ox_ − *E*_red_) between the E─C bond centered oxidation and NIL centered reduction can be determined by cyclic voltammetry and allows for the calculation of the corresponding wavelength related to an intramolecular electron transfer as it is typically done for intermolecular electron transfers in photoredox catalysis.^[^ ^181^ ^]^ 2) TDDFT computations of excited states reveal potential CT character with electron density relocation from the E─C bond to the NIL, which can be visualized by difference density plots. Furthermore, the energy of excited states, which should lay within the visible‐light regime, is obtained. Potential energy surface scans are more sophisticated methods to identify dissociative character of excited states. However, to predict if a main group complex with a given NIL will show photoreactivity based on its Lewis structure alone is still difficult. Even if the above mentioned redox potentials and CT character are promising, the channel branching of excited states is only hardly predictable.

## 4 Recent Developments

### 4.1 Phenalenyldiamines

**Scheme 9:**
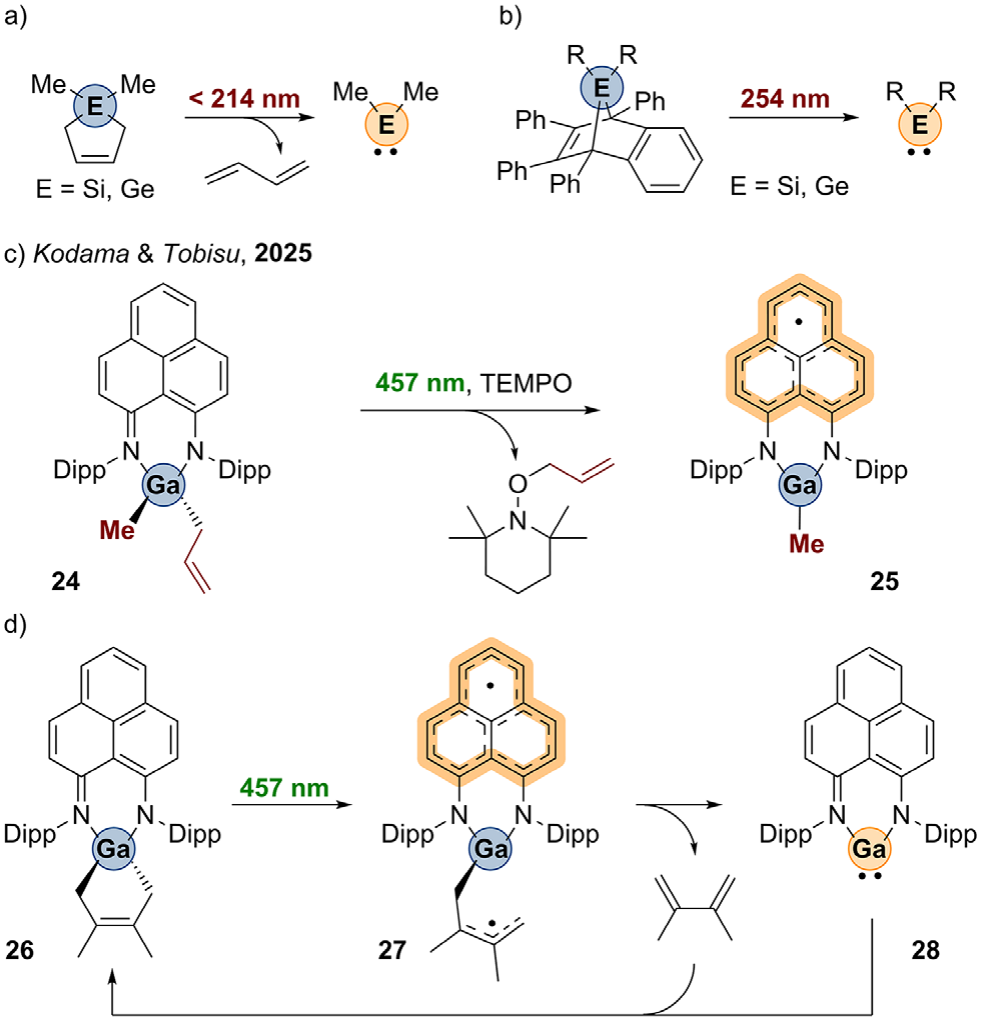
a) and b) UV‐induced [4+2]‐cycloreversion of 3‐silolenes and 3‐germolenes (Si: R = Me; Ge: ‐R = Me, Et, Ph, Mes). c) Ga─C homolysis of **24** by visiblelight. d) Visible‐light‐induced [4+2]‐cycloreversion of **26**.

Just recently, Kodama and Tobisu reported on Ga─C homolysis with 457 nm within the non‐innocent phenalenyldiamine ligand framework.^[^ ^182^ ^]^ The photolysis of the heteroleptic gallium complex **24** allowed for selective generation of an allyl radical, which was trapped by TEMPO. The resulting gallium radical species **25** was characterized as ligand‐centered radical by EPR spectroscopy and supporting DFT calculations (Scheme 9c). The 3‐gallalene **26** undergoes similar Ga─C homolysis by visible‐light irradiation to provide the diradical compound **27** as intermediate finally forming compound **28**, which is a room‐temperature‐stable gallylene (Scheme 9d).^[^ ^183^ ^]^ Reactions like the photolytic cycloreversion of **26** to **28** are known for NIL‐lacking compounds of group 14^[^ ^184^, ^185^ ^]^ and are usually initiated by UV irradiation (Scheme 9a,b).^[^ ^186^, ^187^, ^188^, ^189^, ^190^, ^191^, ^192^, ^193^, ^194^ ^]^ The reductive fragmentation of **26** to **28** illustrates how visible‐light‐induced E─C bond homolysis mediated by NILs enables unusual reversible two‐electron processes within compounds of typically redox‐innocent main group elements.

### 4.2 Bis(pyridylimino) Isoindolides

**Scheme 10:**
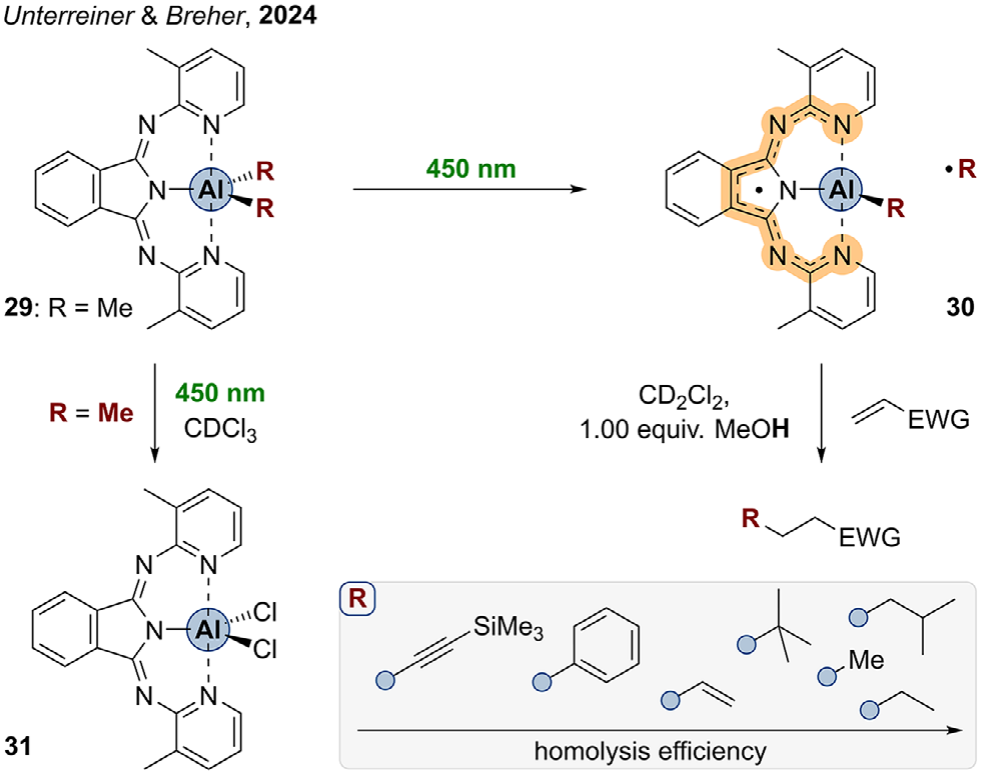
Visible‐light‐induced Al─C bond homolysis of BPI aluminum complexes.

In 2024, our group reported in a collaborative study on aluminum complexes of bis(pyridylimino) isoindolide (BPI) ligands (Scheme 10).^[^ ^195^ ^]^ By irradiation with 450 nm, the dimethyl substituted complex [^Me2^BPI−AlMe_2_] (**29**) undergoes homolytic Al─C bond scission to form the aluminum containing BPI‐centered radical [{^Me2^BPI^•^}−AlMe] (**30**). The formation of methyl radicals was corroborated by radical and spin trapping experiments with DMPO and TEMPO, respectively. Conducted in chloroform, the reaction yielded the chlorinated species [^Me2^BPI−AlCl_2_] (**31**) like in the case of aluminum porphyrins (cf. Scheme 2). Furthermore, the complexes were applied as radical sources in the Giese‐type conjugate addition^[^ ^196^ ^]^ to electron‐deficient alkenes. Those C─C coupling reactions were conducted at ambient conditions and in the presence of one equivalent of methanol as hydrogen atom donor source. This implied that the alkylation is following a radical‐polar‐crossover mechanism, which is enabled by the redox‐activity of the BPI ligand.

**Figure 1:**
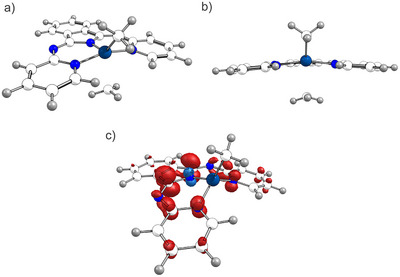
a) Side‐view on the molecular structure of the S_1_ state of **32**. b) Front‐view within the BPI plane. c) Computed molecular structure and spin density plot of the postulated primary photolysis product **30**. Adapted from Reference 195.

The speed of Al─C bond homolysis was described as highly dependent on the degree of hybridization of the carbon moiety paralleling previous findings from porphyrin chemistry (vide supra). An EDA analysis revealed a better orbital interaction of the aluminum atom with orbitals of increasing s‐character as basis for higher BDEs, thus slower homolysis. TDDFT calculations enabled structural characterization of the first excited singlet state S_1_ of the slightly modified aluminum complex [BPI−AlMe_2_] (**32**), which is depicted in Figure 1a,b. Interestingly, one of the Al─C bonds is strongly elongated by 35 pm indicating dissociative nature of the S_1_ state. The former C═N double bonds of the two imine moieties were 3.5 pm longer compared to the electronic ground state geometry. Such bond length alterations were reported as the consequence of raised electron density in the π* levels of BPI ligands.^[^ ^197^ ^]^ Thus, the first optical transition at 450 nm excites the molecule into the first excited singlet state, whereby electron density from the Al─C bond is reorganized into the ligand π system. The intermediate radical [{^Me2^BPI^•^}−AlMe] **30** could not be isolated as the organic radical {R^•^} usually recombines with **30** by ligand alkylation if not trapped by any substrate. Still, computations of this postulated primary photolysis product revealed fully ligand‐centered spin density (Figure 1c) and a distorted tetrahedral coordination environment around Al. The geometric features of the radical homolysis product is already slightly realized in the S_1_ geometry (Figure 1a).

Transient UV–vis absorption spectroscopy revealed longer excited state lifetimes compared to control measurements of the sole BPI ligand. This is supporting a change in excited state dynamics upon introduction of the organometallic fragment. The excited state is depopulated by three different pathways, which are internal conversion, the photoreaction and a third process, most likely an intersystem crossing to the triplet surface. The excited triplet state of **32** showed no dissociative character regarding the Al─C bond featuring in summary homolysis from the S_1_ state as highly likely.

## 5 Conclusion and Outlook

Organometallic compounds capable of E─C bond homolysis are of great interest to chemists due to their applications in polymer chemistry, organic synthesis, and catalysis. Most beneficial are photoinitiators, which can be activated by visible light and are based on earth‐abundant metals such as light main group elements, e.g., aluminum or silicon. This represents a conflict as the E─C homolysis of traditional organometallics of light main group elements requires harsh UV irradiation due to the lack of a chromophore and strong E─C bonds. The presence of redox NILs allowed for homolysis of the E─C bond within multiple compounds with wavelengths in the visible‐light range. What appears to be a general concept is that the bond dissociation energy of the E─C bond is lowered because the radical homolysis product {E^•^} is thermodynamically stabilized by electron density delocalization. In other words, the NIL acts as a photooxidant, which absorbs light in the visible‐light range and is reduced by single‐electron transfer from the E─C bond.

Immediate potential of visible‐light photolysis lays in the utilization of main group compounds as radical sources in radical polymerizations or in homogeneous catalysis. The necessity of stochiometric amounts of complex displays a drawback compared to fields like photoredox catalysis, but otherwise the intramolecular nature of the relevant photophysical process avoids the need for long excited state lifetimes to achieve efficient photoreactivity. If the main group fragment can be incorporated into catalytic cycles after homolysis, transition‐metal like catalysis becomes realistic and the synthetic potential of the title concept seems unlimited. Still, there is a large mismatch between the interest in E─C bond homolysis for applications and the understanding of why and how fast the radical formation occurs. Comprehensive understanding of the connection between molecular structure and photolysis tendencies is not yet established and photoreaction quantum yields still have to be determined experimentally. Apparently, large contributions of the E─C bond to the frontier orbitals are a key factor for successful photolysis, and one accessible excited state has to show charge transfer character, decreasing the electron density within the E─C bond. Currently, there are too few systems accurately mechanistically investigated to ascertain this concept. We want to emphasize that working towards structure–photoreactivity correlations is challenging but will be highly beneficial for the implementation of E─C photolysis in various scientific areas. For this purpose, future studies should probe excited state dynamics by diverse photophysical methods including transient spectroscopy, electrochemical, and state‐of‐the‐art computational methods^[^ ^198^ ^]^ to unravel the full potential of this exciting field of chemistry.

## Conflict of Interests

The authors declare no conflict of interest.

## Data Availability

Data sharing is not applicable to this article as no new data were created or analyzed in this study.

## Footnotes

1 Electromers are isomers with equal constitution but different electron density distribution, which is accompanied by similar but distinct molecular structures with only minuscule differences in the spatial arrangement of atoms, but which are all representing local minima on the potential hypersurface.^[^ ^108^, ^199^, ^200^ ^]^ Within this article, the synonyms “valence tautomers”, “redox isomers”, or “valence isomers” are not used.
